# Thirteen year retrospective review of the spectrum of inborn errors of metabolism presenting in a tertiary center in Saudi Arabia

**DOI:** 10.1186/s13023-016-0510-3

**Published:** 2016-09-15

**Authors:** Majid Alfadhel, Mohammed Benmeakel, Mohammad Arif Hossain, Fuad Al Mutairi, Ali Al Othaim, Ahmed A. Alfares, Mohammed Al Balwi, Abdullah Alzaben, Wafaa Eyaid

**Affiliations:** 1 Department of Pediatrics, King Abdulaziz Medical City, Riyadh, Saudi Arabia; 2 King Saud bin Abdulaziz University for Health Sciences, King Abdulaziz Medical City, Riyadh, Saudi Arabia; 3 Ministry of National Guard-Health Affairs (NGHA), Riyadh, Saudi Arabia; 4 Department of Pathology, King Abdulaziz Medical City, Riyadh, Saudi Arabia; 5 Qassim University, College of Medicine, Department of Pediatrics, Almulyda, Saudi Arabia; 6 Division of Genetics, Department of Pediatrics King Saud bin Abdulaziz University for Health Sciences, King Abdulaziz Medical City, Riyadh, PO Box 22490, 11426 Saudi Arabia; 7 Advanced Clinical Research Center, Shin-Yurigaoka General Hospital, Kawasaki, Kanagawa Japan

**Keywords:** Inborn errors of metabolism, IEMs, Saudi Arabia, Lysosomal, Organic acidemia, Mitochondrial, Fatty acid oxidation defects

## Abstract

**Background:**

Inborn errors of metabolism (IEMs) are individually rare; however, they are collectively common. More than 600 human diseases caused by inborn errors of metabolism are now recognized, and this number is constantly increasing as new concepts and techniques become available for identifying biochemical phenotypes. The aim of this study was to determine the type and distribution of IEMs in patients presenting to a tertiary care center in Saudi Arabia. METHOD: We conducted a retrospective review of children diagnosed with IEMs presenting to the Pediatric Department of King Abdulaziz Medical City in Riyadh, Saudi Arabia over a 13-year period.

**Results:**

Over the 13- year period of this retrospective cohort, the total number of live births reached 110,601. A total of 187 patients were diagnosed with IEMs, representing a incidence of 169 in 100,000 births (1:591). Of these, 121 patients (64.7 %) were identified to have small molecule diseases and 66 (35.3 %) to have large molecule diseases. Organic acidemias were the most common small molecule IEMs, while lysosomal storage disorders (LSD) were the most common large molecule diseases. Sphingolipidosis were the most common LSD.

**Conclusion:**

Our study confirms the previous results of the high rate of IEMs in Saudi Arabia and urges the health care strategists in the country to devise a long-term strategic plan, including an IEM national registry and a high school carrier screening program, for the prevention of such disorders. In addition, we identified 43 novel mutations that were not described previously, which will help in the molecular diagnosis of these disorders.

## Background

Inborn errors of metabolism (IEMs) are defined as monogenic diseases that result in dysfunctional proteins encoded by different genes, which in many cases lead to loss of activity of the enzymes involved [1]. More than 600 different inborn errors of metabolism have been recognized up to this point, and this number is constantly increasing as new concepts and techniques become available for identifying biochemical phenotypes. IEMs are extremely heterogeneous making their classification a primary challenge. Several informal systems of classification currently exist. IEMs can be classified according to the organs involved, such as neurological or hepatic disorders or according to the organelle involved, such as mitochondrial, peroxisomal or lysosomal disorders. Disorders can also be classified according to the age of presentation ranging from neonatal onset to juvenile and adult onset. Because each of these approaches may depend upon the actual setting, no single classification is universally applied [2]. One common and informative classification system involves the classification of IEMs into small and large molecule disorders [3]. Small molecule IEMs have an acute intoxication presentation with a remitting-relapsing clinical course. These include organic acidemias, vitamin responsive disorders, urea cycle disorders, inborn errors of carbohydrates, haem synthesis defects, cholesterol biosynthesis defects, and amino acids and metal transport defects. Large molecule IEMs have a gradual and insidious progressive presentation. These include glycogen storage disorders, sphingolipidosis, mucopolysaccharidosis, oligosaccharidosis, mitochondrial disorders and congenital disorders of glycosylation [2]. The diagnosis of IEMs is mainly based on biochemical investigations, which include the screening of several metabolites in the blood, urine and cerebrospinal fluid (CSF); analysis of enzymatic activities, and molecular genetics testing.

The incidence of such disorders vary from country to country and from region to region. In one Australian study the incidence was reported to be 15.7 per 100,000 births whereas, in Italy, the reported incidence was 27 per 100,000 births [4, 5]. In the West Midlands region in the United Kingdom (UK), the incidence reached up to 1:784 and in British Columbia, Canada, the incidence was reported to be 1:2500 [6, 7].

These disorders are usually inherited as autosomal recessive disorders, explaining why IEMs are common in populations with a high rate of consanguineous marriages, such as Saudi Arabia. In Saudi Arabia, the rate of consanguineous marriages reaches up to approximately 60 % [8, 9].

Despite the high frequency of IEMs, apart from the eastern region study [10], only a few anecdotal epidemiological studies in Saudi Arabia have discussed the incidence, type and distribution of such devastating disorders. Most of the remaining reports in the literature were limited to case reports and case series. In this study, we report the incidence, type, and distribution of IEMs presenting to King Abdulaziz Medical City (KAMC) in the middle region of Saudi Arabia over 13 years. In addition, we identified 43 novel mutations.

## Methods

King Abdulaziz Medical City (KAMC) is a tertiary center in Saudi Arabia, that commenced operations in 1983. The bed capacity is 715, and an average of 8500 births per year occur at the center. This medical city passed the requirements for accreditation under the Joint Commission International (JCI) standards with excellent performance in December 2006. The current study is a retrospective review of all cases at the Pediatric Department of King Abdulaziz Medical City (KAMC) in Riyadh, Saudi Arabia. The duration of the study was 13 years, from 01-01-2001 to 31-12-2014. All patients included were born during this period. The cases were identified according to medical records via diagnostic codes and the genetic division’s database. The study was approved by the research committee of King Abdullah International Medical Research Centre (KAIMRC) in Riyadh, Saudi Arabia.

The diagnostic algorithm starts by referring patients based on clinical suspicion to genetic/metabolic facility from other departments, and from positive new born screening (NBS) after 2011. All accepted cases undergo phenotype related screening biochemical investigations. Confirmation of diagnosis is then achieved either by measuring enzyme activity and/or by targeted molecular tests. In case of unrevealing confirmatory investigations or vague presentation, whole exome or whole genome sequencing is requested.

The diagnosis of each IEM was based on clinical and biochemical investigations, including analyses of ammonia, lactic acid, total homocysteine, plasma amino acids, the acylcarnitine profile, urine aminoacids, urine for organic acids, copper, ceruloplasmine levels, very long chain fatty acids, transferrin isoelectrofocusing, carbohydrate-deficient transferrin and urine for polyols.

DNA molecular genetic testing was performed in commercial clinical international labs including CENTOGENE, GeneDx, Emory Genetics, Cincinnati Children’s Hospital Medical Center, Bioscientia and Nijmegen Medical Center. All of the parents of the patients with IEMs were tested for carrier status.

Incidence was calculated by dividing the number of diagnosed cases by the number of total births during the study period and multiplying by 100,000 [11].

## Results

Over the 13-year period of this retrospective cohort study, the total number of live births reached 110,601. A total of 187 patients were diagnosed with an IEM, resulting in an incidence of 169 in 100,000 births (1:591). Of these patients, 121 (64.7 %) were identified to have a small molecule disease (Table 1) and 66 (35.3 %) to have a large molecule disease (Table 2). The overall mean, median and range of age at diagnosis were 3.2 years, 1.2 years and from 1 day to 13 years respectively. Tables 1 and 2 show the type and distribution of IEMs from 2001 to 2014 and illustrate the estimated incidence per 100,000 live births for each group of disorders and the mean, median and range of age at diagnosis for each group. The lysosomal storage disorders (LSDs) were the most common diagnosed group, in general, and were observed in 39/187 patients (20.8 %). Sphingolipidoses represent the largest subgroup of the LSDs (22/39; 56.4 %), and GM1 gangliosidosis (infantile phenotype) was the most prevalent disorder. The second most common category was organic acidemias (34/187; 18.2 %), with propionic acidemia (PA) as the most common disorder in that group. Aminoacidopathies were diagnosed in 30/187 patients (16 %). Fatty acid oxidation defects (FAOD) were diagnosed in 5/187 patients (2.7 %), and the frequency of patients with urea cycle disorders (UCD) was 12/187 (6.4 %). The most common FAOD was very long-chain acyl-CoA dehydrogenase deficiency (VLCAD), while argininosuccinic aciduria was the most common UCD. Mucopolysaccharidoses (MPS) were diagnosed in 15/187 patients (8 %), with MPS VI as the predominant type. Fourty-three novel mutations were identified, and missense mutations were the most common type of mutations (Tables 3 and 4). All the listed novel mutations fit with the clinical features of the disease, biochemical biomarkers support genotype phenotype correlation and they segregate well within the patients and family members.

**Table 1:** Small-molecule disorders of IEMs in KAMC (2001–2014). Total numbers of live births (110,601)

Disease category	Number of cases diagnosed	Incidence per 100,000	Mean age at diagnosis	Median age at diagnosis	Range of age
Organic acidemias	34	30	1.8 years	60 days	1 day–10 years
Propionic acidemia	9		30.2 days	20 days	1 day–6 months
Methylmalonic acidemia	7				
Mutase deficiency	5				
Cobalamin A defect	1				
Cobalamin C defect	1				
Glutaric acidemia	3				
3-hydroxy-3-methylglutaryl-CoA lyase deficiency	4				
3-Methylcrotonylco A carboxylase deficiency	3				
Biotinidase deficiency	3				
3-Methylglutaconic Aciduria Type III	1				
Ethylmalonic encephalopathy	1				
B-ketothiolase deficiency	1				
Isovaleric acidemia	1				
Malonic aciduria	1				
Aminoacidopathies	30	27	3.3 years	10.5 months	1 day–13 years
Homocystinuria	14		7 years	7.5 years	
• Classical	11				
• MTHFR deficiency	2				
• MAT deficiency	1				
PKU	5				
• Classical	3				
• Non-PKU hyperphenylalaninemia	2				
Biopterin Synthesis Defect PTPS deficiency	4				
MSUD	5				
Asparagine synthetase deficiency	2				
Vitamins responsive disorders	18	16	5.7 years	5.5 years	6 months–10 years
Biotin Thiamine Responsive Basal Ganglia Disease	17				
Pyridoxine-dependent epilepsy	1				
Inborn Errors of Carbohydrates	12	11	3.1 years	1.3 years	1 week–7 years
Galactosemia	4				
Transaldolase deficiency	6				
Hereditary fructose intolerance	1				
Fructose 1,6 bisphosphatase deficiency	1				
Urea Cycle Disorders	12	11	12 days	7 days	1 day–30 days
Argininosuccinic Aciduria	8				
Citrullinemia	4				
Fatty Acid Oxidation Defects	5	4	1.4 years	2 days	2 days–7 years
VLCAD deficiency	3		21 days	2 days	2 days–60 days
MCAD deficiency	1		2 days	2 days	2 days
Carnitine uptake defect	1		7 years	7 years	7 years
Aminoacids transport defect	5	4	10 years	11 years	6–13 years
Cystinuria	5				
Metal transport defect	2	2	8.5 years	8.5 years	7–10 years
Wilson disease	2				
Disorders of Haem biosynthesis	2	2	12.5 years	12.5 years	12–13 years
Acute intermittent porphyria	2				
Cholesterol biosynthesis defect	1	1	1 year	1 year	1 year
CHILD syndrome	1				
Total	121	109	3.3 years	9 months	1 day–13 years

*MTHFR* methylenetetrahydrofolatereductase, *MAT* methionine adenosyltransferase, *PKU* phenylketonuria, *MSUD* maple syrup urine disease, *VLCAD* very long-chain acyl-CoA dehydrogenase, *MCAD* medium-chain acyl-CoA dehydrogenase, *CHILD* Congenital hemidysplasia with ichthyosiform erythroderma and limb defects, *PTPS* 6-Pyruvoyl-Tetrahydropterin Synthase

**Table 2:** Large-molecule disorders of IEMs in KAMC (2001–2014). Total numbers of live births (110,601)

Disease category	Number of cases diagnosed	Incidence per 100,000	Mean age at diagnosis	Median age at diagnosis	Range of age
Lysosomal Storage Diseases (LSD)	41	37	3.6 year	3 years	2 months–13 years
Sphingolipidosis	22	20	3.1 years	2 years	2 months–13 years
Fabry disease	3				
Sandhoff disease	2				
Niemann–Pick disease type B	1				
Niemann–Pick disease type C	3				
GM1 gangliosidosis (infantile phenotype)	4				
Metachromatic leukodystrophy	3				
Saposin B Deficiency	2				
Krabbe disease	1				
Mucopolysaccharidosis (MPS)	15	14	5 years	5 years	5 months–12 years
MPS I	1				
MPS II	1				
MPS IIIA	2				
MPS IVA	5				
MPS VI	6				
Oligosaccharidosis	2	2	3 years	3 years	2–4 years
Mucolipidosis II	1				
α-mannosidosis	1				
Others					
Neuronal ceroid-lipofuscinoses	3: 2 type 6, and 1 type 8		5.3	5	5–6 years
GSD II	2		3.1 months	3.1 months	1 week to 6 months
Glycogen storage diseases (GSD)	5	4	2.2 years	2 years	15 months–4 years
GSD III	1				
GSD IV	1				
GSD IX	3				
Mitochondrial disorders	12	11	2.2 years	8 months	1 week–8 years
Leigh disease	3				
Pyruvate dehydrogenase deficiency	2				
Pyruvate carboxylase deficiency	2				
Mitochondrial DNA depletion syndrome 3	1				
Mitochondrial DNA depletion syndrome 5	1				
Cardioencephalomyopathy, fatal infantile, due to cytochrome c oxidase deficiency 1	1				
3-Methylglutaconic aciduria with deafness, encephalopathy, and Leigh-like	1				
Primary Coenzyme Q10 deficiency type 5	1				
Peroxisomal disorders	7	6	2 years	9 months	1 week–8 years
Primary hyperoxaluria type 1	5				
Zellweger syndrome	1				
Rhizomelic Chondrodysplasia Punctata	1				
Congenital disorders of glycosylation (CDG)	1 (CDG 1 L)	1	8 years	8 years	8 years
Total	66	60	3.1 years	2 years	1 week–13 years

**Table 3:** Mutations for small molecule IEMs

Disease category	Disease	Gene	Reported mutations	Novel mutations	Founder Vs. Private	Type of mutation
Organic acidemias	Propionic acidemia	*PCCA*		c.425G > A(p. Gly142Asp)	Founder	Homozygous, missense
			c.350G > A (p.Gly117Asp)		Private	
		*PCCB*	c.1050dupT		Private	Dupplication
	Methylmalonic acidemia	*MUT*		c.329 A > G(p. Tyr110Cys)	Founder	Homozygous, missense,
			c.1677-1G > C		Private	Splice
	Cobalamin A Defect	*MMAA*	c.586C > T (p.Arg196*)		Private	Nonsense
	Cobalamin C defect	*MMACHC*	c.394C > T (p. Arg132*)		Private	Nonsense
	Glutaric acidemia	*GCDH*	c.1144G > A (p.Ala382Thr)		Private	missense
				c.853-2A > G (IVS8-2A > G)	Private	Splice
				c.278A > G (p.His93Arg)	Private	missense
	3-hydroxy-3-methylglutaryl-CoA lyase deficiency	*HMGCL*	c.122G > A		Founder	missense
	3-Methylcrotonyl CoA carboxylase deficiency	*MCCC1*		c.1808 dup A(p. p.Asn603 Lysfs*5)	Private	Homozygous, duplication
		*MCCC2*	c.1147A > T (p.Lys383*)		Private	Nonsense
	Biotinidase deficiency	*BTD*	c.755A > G (p.Asp252Gly) c.1330G > C (p.Asp444His)		Private	Two heterozygous missense mutations in Exon 4
	3-Methylglutaconic aciduria type III	*OPA3*		c.194delG(p. Gly65Alafs*7)	Private	Homozygous, deletion
	Ethylmalonic encephalopathy	*ETHE1*		c.263 C > T(p. Ser88Leu)	Private	Homozygous, missense
	B-Ketothiolase deficiency	*ACAT1*		c.412-419del(p. Gln138Tyrfs*36)	Private	Homozygous, deletion
	Isovaleric acidemia	*IVD*		c.358C > T(p. Arg120X)	Private	Homozygous, nonsense
	Malonyl-CoA decarboxylase deficiency	*MLYCD*		c.953_954delAG(p. Glu318Valfs*35)	Private	Homozygous, deletion
Aminoacidopathies	Homocystinuria					
	Classical	*CBS*	c.969G > A (p.Trp323Ter)		Founder	Homozygous missense
			c.1006C > T (p.Arg336Cys)		Founder	Homozygous missense
	MTHFR deficiency	*MTHFR*	c.680C > T (p.Thr227Met)		Private	
	MAT deficiency	*MAT1A*		c.1081G > T(p.Val361Phe)	Private	Homozygous, missense
	PKU	*PAH*	c.1169A > G (p.Glu390Gly)		Private	Homozygous, missense
	PTPS deficiency	*PTPS*		c.238A > G(p. Met80Val)	Founder	Homozygous, missense
			c.169_171delGTG (p.Val57del)		Founder	Homozygous, deletion
	MSUD	*BCKDHA*		c.347A > G(p. Asp116Gly)	Private	missense
			c.905A > C (p.Asp302Ala)		Founder	missense
		*BCKDHB*		c.674 T > C(p.Leu225Pro)	Private	missense
				c.1144 T > C(p.Cys382Arg)	Private	Homozygous, missense
	Asparagine synthetase deficiency	*ASNS*		c.1160A > G (p.Tyr377Cys)	Founder	Homozygous, missense
Vitamins responsive disorders	Biotin Thiamine Responsive Basal Ganglia Disease	*SLC19A3*	c.1264A > G (p.Thr422Ala)		Founder	Homozygous, missense
	Pyridoxine-dependent epilepsy	*ALDH7A1*	c.877dupAA (p.Ser293Lysfs*22)		Private	Duplication
Inborn Errors of Carbohydrates	Galactosemia	*GALT*	c.691 C > T (p.Arg231Cys)		Founder	Homozygous, missense
			c.404C > T (p.Ser135Leu)		Private	Homozygous, missense
			c.563A > G (P.Gln188Arg)		Private	Homozygous, missense
	Transaldolase Deficiency	*TALDO1*	c.793delC (p.Gln265ArgfsX56)		Founder	Deletion
	Hereditary fructose intolerance	*ALDOB*	c.360_363delCAAA (p.Asn119LysfsX31)		Private	Deletion
	Fructose 1,6 bisphosphatase deficiency	*FBP1*	c.114_119dup (p. Cys39_Thr40dup)		Private	Duplication
Urea cycle disorders	Argininosuccinic Aciduria	*ASL*	c.556C > T (p.Arg186Trp)		Founder	Missense
			c.1060C > T (p.Q354X)		Founder	Nonsense
	Citrullinemia type 1	*ASS1*		c.364-2A > G	Founder	Homozygous, intronic
			c.370G > A (p.Asp124Asn)		Founder	Homozygous, missense
Fatty acid oxidation defect	VLCAD	*ACADVL*		c.494 T > C(Phe165Ser)	Private	Homozygous, missense
	VLCAD	*ACADVL*	c.65C > A (p.Ser22*)		Founder	Nonsense
	MCAD	*ACADM*		c.255 G > T(p.Gly119*);)	Private	Homozygous, nonsense
				c.938 T > G(p.Phe313Cys	Private	Homozygous, missense
	Carnitine uptake defect	*SLC22A5*		c.1385G > A(p. Gly462Asp)	Private	Homozygous, missense
Aminoacids transport defect	Cystinuria	*SLC3A1*		c.1711 T > A(p.Cys571Ser)	Founder	Homozygous, missense
			c.1400 T > A (p.Met467Lys)		Private	
		*SLC7A9*		c.1166 C > T(p.Thr389Met)	Private	Homozygous, missense
Metal Transport Defect	Wilson disease	*ATP7B*	c.2230 T > C (p.Ser744Pro)		Founder	Homozygous, missense
Disorders od Haem biosynthesis	Acute Intermittent Poephyria	*HMBS*	c.760delC (p.Leu254X)		Founder	Nonsense
Cholesterol biosynthesis defect	CHILD syndrome	*NSDHL*	c.314C > T (p.Ala105Val)		Private	Homozygous, missense

*PKU* phenylketonuria, *MSUD* maple syrup urine disease, *VLCAD* very long-chain acyl-CoA dehydrogenase, *MCAD* medium-chain acyl-CoA dehydrogenase

**Table 4:** Mutations for large molecule IEMs

Disease category		Disease	Gene	Reported mutations	Novel mutations	Founder Vs. Private	Type of mutation
LSD	Sphingolipidosis	Fabry	*GLA*	c. 782G > T (p.Gly261Val)		Founder	Homozygous, missense
		Sandhoff disease	*HEXB*		c.1169 + 3_1169 + 10delAAGTTGTT (p.Gly65 AlafsX7)	Private	Deletion
		Niemann-Pick disease type B	*SMPD1*	c.1267 C > T (p.His423Tyr)		Founder	Homozygous, missense
		Niemann-Pick disease type C	*NPC1*		c.2130 + 1G > A;	Founder	Homozygous, intronic
					c.2443_2444delp.ser815Leufs*54	Private	deletion
		GM1 gangliosidosis	*GLB1*		c.950G > A(p. Trp317*)	Private	Homozygous, nonsense
				c.171C > G (p.Tyr57X)		Founder	Homozygous, missense
		Metachromatic leukodystrophy	*ARSA*		c.1108-2A > G	Private	Homozygous, intronic
		Saposin B deficiency	*PSAP*	c.722G > C (p.Cys241Ser		Founder	Homozygous, missense
		Krabbe disease	*GALC*		c.396G > A(p.Trp132*)	Private	Homozygous, nonsense
	Mucopolysaccharidosis (MPS)	MPSI	*IDUA*		c.1868 T > C(p. Leu623Pro)	Private	Homozygous, missense
		MPSII	*IDS*		c.405A > C(p. Lys135Asn)	Private	Homozygous, missense
		MPSIIIA	*SGSH*		c.664-13C > G	Private	Homozygous, intronic
				c.535G > A (p.Asp179Asn)		Private	Homozygous, missense
		MPS IVA	*GALNS*	c.120 + 1G > C (IVS1 + 1G > C)		Private	Homozygous, missense
				c.860C > T (p.Ser287Leu)		Private	Homozygous, missense
				c.697G > A (p.Asp233Asn)		Private	Homozygous, missense
		MPSVI	*ARSB*	c.753C > G (p.Tyr251*)		Founder	Homozygous, nonsense
				c.430A > G (p.His393ARG)		Founder	Homozygous, missense
				c.1079 T > C (p. Leu360Pro)		Private	Homozygous, missense
	Oligosaccharidosis	Mucolipidosis II	*GNPTAB*	c.3503_3504 delTC (p.Phex1172)		Private	Homozygous, deletion
		α-mannosidosis	*MAN2B1*	c.1340A > T (p.Asp447Val)		Private	Homozygous, missense
	Others	NCL type 6	*CLN6*		c.794_796del(p.Ser265del)	Private	Homozygous, deletion
				c.794_796delCCT		Private	Homozygous, deletion
		NCL type 8	*CLN8*	Homozygous deletion encompassing exon2		Private	Homozygous, deletion
		GSDII	*GAA*		c.1431delT(p. lle477fs)	Private	Homozygous, deletion
					c.1657C > T(p. Gln553*)	Private	Homozygous nonsense
Glycogen storage disease		GSDIII	*AGL*		c.4353G > T(p. Trp1451Cys);	Private	Homozygous, missense
		GSDIV	*GBE1*		c.998A > T (p.Glu 333 Val)	Private	Homozygous, missense
		GSD IX	*PHKG2*	c.130C > T (p.Arg44*)		Founder	Homozygous nonsense
			*PHKB*		Deletion Exon 5 and 6	Private	Homozygous, deletion
Mitochondrial disorders		Leigh disease	*MTATP6*	m.8993 T > G (p.Leu156Arg)		Private	Homoplasmic, missense
			*COX15*	c.649C > T (p.Arg217Trp)		Private	Homozygous, missense
		Pyruvate dehydrogenase deficiency	*PDHA1*	c.1256_1259dup (p.Trp421Serfs*6)		Private	Heterozygous Duplication
			*PDHA1*		c.1132C > T (p.Arg378Cys)	Private	Hemizygous missense
		Pyruvate Carboxylase Deficiency	*PC*		c.3116_3126del (p.Leu1039Glnfs*7)	Private	Deletion
		Mitochondrial DNA depletion syndrome 3	*DGUOK*	c. 617G > A (p. R206k)		Private	Homozygous, missense
		Mitochondrial DNA depletion syndrome 5	*SUCLA2*		c.362_363del (p.Ile121Serfs*38)	Private	Deletion
		Cardioencephalomyopathy, fatal infantile, due to cytochrome c oxidase deficiency 1	*SCO2*	c.2 T > C(p.Met1?)		Private	Homozygous, missense
		3-Methylglutaconic aciduria with deafness, encephalopathy, and Leigh-like	*SERAC1*	c.438del (p.Thr147Argfs*22)		Private	Deletion
		Primary CoenzymeQ10 deficiency type 5	*COQ9*		chr16_57485062C > T (p.His62Arg)	Private	Homozygous, missense
Peroxisomal disorders		Primary hyperoxaluria type 1	*AGXT*	c.187G > C (p.Gly63Arg)		Founder	Homozygous, missense
		Zellweger syndrome	*PEX5*	c.1578 T > G (p.Asn526Lys)		Private	Homozygous, missense
		Rhizomelic chondrodysplasia punctata type 1	*PEX7*		c.321_322delTA(p.Tyr107*)	Private	Homozygous, deletion
Congenital disorder of glycosylation (CDG)		CGD 1 L	*ALG9*	c.1075G > A (p.E359K)		Private	Homozygous, missense

## Discussion

In this report, we describe the incidence of IEMs in a single tertiary center in the middle region of Saudi Arabia over more than a decade. We reported an incidence of 1:591 individuals, which is the highest incidence for IEMs reported to this point. Our study is the second epidemiological report after that of Moammar et al., 2010, who reported a incidence of 1:667 [10]. If we combine the two studies, the cumulative incidence is 1:635 births or 157 per 100,000, which is still one of highest reported incidence rates across the world. KAMC is one of the largest medical institutions in the Kingdom and is also a referral center; therefore to obtain a more accurate estimation of incidence of these disorders we have excluded the 69 patients who were diagnosed with IEM but not born at KAMC. If we combine the referred cases with those of patients born at the hospital, we obtain an incidence of 231.5 in 100,000 births (1:432).

Our study confirms the conclusions drawn by Moammar et al., 2010, LSDs are the most commonly identified group of disorders and organic acidemias are the most prevalent small molecule diseases. Our work also supports the notion that MPS VI is the most common type of mucopolysaccharidosis in Saudi Arabia. The results also support the wide variation in incidence of genetic diseases between Saudi Arabia and other parts of the world [12]. In our study for example the recorded number of PTPS exceeds the classical PKU which is reverse in Caucasian population [13]. This is more evident in PA, in which incidence in Saudi Arabia far exceeds the global incidence [14]. During the course of our study we discovered 9 new PA cases in our center, with incidence of 1 per 12,500 live births.

Our study showed unique phenotypes in comparison with the literature. Our three VLCAD patients for example had early presentation with severe phenotype ended with early death. Their genotype mutations were one novel missense mutation in exon 6, c.494 T > C (p.Phe165Ser) and the other two had previously reported nonsense mutation in exon 2, c.65C > A (p.Ser22*). Although these mutations are not clear null mutations [15], they resulted in severe phenotype. Alternatively, the most common phenotype in VLCAD is the milder late onset with the missense mutation p.Val283Ala being the most prevalent disease causing variant [15]. This reflect the poor genotype-phenotype correlation.

In this study the number of private mutations was almost double that of founder mutations, which support the previous report of Al-Owain et.al (2012) who noted that private mutations outweigh founder mutations in Saudi Arabia [12].

Interestingly, among the diseases discovered by our study, 35 diseases are amenable for treatment. Recent advances in early diagnostic tools like the expanded New Born Screening program list, which can detect 14 of the listed diseases, and the availability of treatment options like enzyme replacement therapy, opened new horizons for these patients and their families.

With the implementation of next generation sequencing (WES and WGS) we were able to solve many obscure cases. Additionally, new diseases and new variants might be discovered more easily. It is possible to see NGS a first line diagnostic tool in the near future.

The markedly high numbers of metabolic diseases in Saudi Arabia in general and in our center in particular, with mostly homogenous genotypes, paves the way for future collaboration with international parties, research centers and drug industry, to help providing treatment for our patients. This environment is ideal for wide spectrum of clinical trials of various phases, to speed up the process of new medications discovery.

The limitations of the presented cohort study are clear. These include the fact that the study is confined to a single center in one particular region and contains a small sample size; therefore, the internal and external validity of the study is threatened. Such a retrospective review increases the risk of selection and information biases. Therefore, the numbers mentioned in this study should be taken with caution until further larger studies confirm or refute such findings. The retrospective nature risks missing cases due to poor documentation. In addition, some patients with metabolic disorders may not be seen at the medical center due to early death prior metabolic intervention. In addition, cases with a relatively mild disease may never have presented to the specialized metabolic center, which also contributes to the bias inherent in this study. The variability in ages at diagnosis is attributed to the delay in referring cases to the metabolic facility from other departments, and these numbers should not reflect the expected age of presentation for the listed diseases.

The incredibly high rate of IEMs in Saudi Arabia compels the health care administration in the country to develop a long-term strategic plan for the prevention of such disorders. First, a national registry should be implemented, and through that registry, a determination of the most common IEM and most common mutations in the population can be made. Second, genetic screening of high school students by DNA molecular testing should be performed to identify carriers for the most common disorders in the Saudi population. Premarital molecular screening can help couples, who carry the same disease causing variants, to take informed decision regarding their marriage and the consequences of their decision. Such a strategy has proven to be effective in another population [16]. Finally, intensive educational campaigns aimed at the community through schools, TV, and web-based social media should be initiated.

## Conclusion

In this study, we report the incidence, type, and distribution of IEMs presenting to King Abdulaziz Medical City (KAMC) in the middle region of Saudi Arabia over 13 years. We also identified 43 novel mutations in 37 genes. Our study emphasizes the high incidence of IEMs in the Saudi population and urges the health care administration in the country to develop a long-term strategic plan for the prevention of such disorders, including an IEMs national registry and a high school carrier screening program.

## Abbreviations

CHILD: Congenital hemidysplasia with ichthyosiform erythroderma and limb defects
CSF: Cerebrospinal fluid
IEM: Inborn error of metabolism
IEMs: Inborn errors of metabolism
IRB: Institutional review board
JCI: Joint Commission International
KAIMRC: King Abdullah International Medical Research Centre
KAMC: King Abdulaziz Medical City
LSD: Lysosomal storage disorders
MAT: Methionine adenosyltransferase
MCAD: Medium-chain acyl-CoA dehydrogenase
MSUD: Maple syrup urine disease
MTHFR: Methylenetetrahydrofolate reductase
PKU: Phenylketonuria
SCAD: Small-chain acyl-CoA dehydrogenase
UCD: Urea cycle disorders
UK: United Kingdom
VLCAD: Very long-chain acyl-CoA dehydrogenase
